# A New Plant Active Polysaccharide from *Nicotiana* Improves the Lead-Led Impairment of Spatial Memory in Mice by Modulating the Gut Microbiota and IL-6

**DOI:** 10.3390/foods13193177

**Published:** 2024-10-06

**Authors:** Ruili Yang, Feng Zhu, Wanying Mo, Huailong Li, Dongliang Zhu, Zengyang He, Xiaojing Ma

**Affiliations:** 1 School of Food and Bioengineering, Hefei University of Technology, Hefei 230000, China; young02202@outlook.com (R.Y.); zf1764230916@163.com (F.Z.); momomorwin@163.com (W.M.); huaijin180910@163.com (H.L.); 2 Anhui Provincial Key Laboratory of Aerosol Analysis, Regulation and Biological Effect, Hefei 230000, China; zhudl1980@163.com

**Keywords:** active plant polysaccharide, spatial memory, gut microbiota, IL-6, gut–brain axis

## Abstract

Active polysaccharides from plants are broadly applied in the food and health industry. The purpose of this study is to identify a new plant active polysaccharide and to investigate its role in modulating spatial memory. Ultrasonics and DEAE-52 chromatography were used to separate and purify the plant active polysaccharide (PAP). Mice were exposed to 100 ppm of lead acetate from birth to 7 weeks old to establish the memory impairment model. PAPs with concentrations of 200 or 400 ppm were fed to the subject mice each day after weaning in a spatiotemporally separated fashion. At the end of the intervention, mice were examined using the Morris water maze test, microbiome sequencing, cytokine profiling and protein analysis. The derived active polysaccharide was constituted by β-anomeric carbon, indicating a new form of PAP. The PAP significantly ameliorates the memory impairment caused by postnatal lead exposure, as evidenced by the preferred coverage of the test mouse in the hidden platform, demonstrating salient neuroregulatory activity. In terms of the gut microbiome in response to PAP treatment, it was found that the 400 ppm PAP reversed the gut dysbiosis, producing a comparable structure to the intact animals, represented by the relative abundance of Firmicutes and Muribaculum, Desulfovibrio, etc. For cytokines, the PAP reversed the plasma levels of IL-6, suggesting an anti-inflammatory trend in the context of proinflammation caused by lead invasion. By injecting an IL-6 antagonist, Tocilizumab, into the deficient mice, the spatial memory was significantly repaired, which demonstrates the central roles of IL-6 in mediating the positive effect of the PAP. Finally, a histone modification mark, H3K27me3, was found to be potent in responding to the signals conveyed by the PAP. The PAP could improve the memory deficits by remodeling the gut–brain axis centered at the microbiota and IL-6, which is regarded as an important cytokine-modulating brain activity. This is an intriguing instance linking neuromodulation with the active polysaccharide, shedding light on the innovative applications of plant polysaccharides due to the scarcity of similar phenotypic connections.

## 1. Introduction

Recently, a collection of active polysaccharides from plants were found to possess health-promoting effects [1,2,3,4], as represented by antioxidative activities [5,6,7]. For instance, a polysaccharide from the *Ophiopogon japonicus* displayed antioxidative and immunological activities and thus was explored as a new form of functional food [8], and a novel polysaccharide (PL-A11) purified from *Phellinus linteus* showed robust antioxidative activities and could be employed to reduce the malondialdehyde level in aged mice [9]. Although many plant polysaccharides showed anti-inflammatory properties, no similar effect was identified in *Nicotiana* polysaccharides. Furthermore, their implication in brain-related pathology has never been established, despite the fact that a novel polysaccharide isolated from Durian seed showed protective effects against Alzheimer’s disease in a transgenic Caenorhabditis elegans model [10].

*Nicotiana* is an annually grown herbaceous plant, and its bioactive substances have long been considered to have great economic and social values [11,12,13]. Compared to other biological substances in this plant [14,15], the roles of active polysaccharides are still not fully understood. In this context, a limited amount of substances were previously isolated from *Nicotiana* [16,17]. Some of the polysaccharide-like ingredients were revealed to show antioxidant activities based on in vitro experiments [17], in the absence of associations with animal or human studies. Due to the limited number of species, the heath-modulatory effect of these polysaccharides remained unknown, leaving their potential value an open question.

The gut microbiota is composed of over 1000 microbial species and 100-fold more genes than the human genome; it is a community tightly linked to various aspects of health [18,19], including brain activity. The microbiota–gut–brain (MGB) axis is the route by which the gut microbiota exerts an effect on the central nervous system, thereby projecting the neuroregulatory function [5,20]. It was found that some plant polysaccharides could alleviate the symptoms of neurological disorders by impacting the gut microbiota [21,22], thus allowing them to act as bioactive substances with MGB-modulating activity. As a recent example [21], polysaccharide extracts (PEs) of *Schisandra chinensis* mitigated depressive-like behaviors by regulating the MGB axis, in which the anti-inflammatory activity of PEs played an essential role. To date, no link between the plant active polysaccharide (PAP) from this species and the gut–brain axis has been proposed.

To this end, the present study aims to purify a new species of PAP and further investigate its roles in modulating brain behaviors. To achieve this, a lead-exposed paradigm was constructed to manifest a memory impairment model, which was generally used to represent the physiological severity caused by widespread and long-term lead exposure [23]. Furthermore, an isolated PAP was administered to the subject mice, and their memory behaviors, gut microbiota, intermediary immune pathways, cerebral molecules, and relations were subsequently examined. This study is a first example of the use of PAPs to modulate brain behavior and the gut–brain axis, shedding light on the trans-systemic nutritional expansion and mechanistic insight of active polysaccharides.

## 2. Materials and Methods

### 2.1. Extraction and Purification of Active Polysaccharide

The extraction of the polysaccharide was performed as described previously [24] with some modifications. First, 1 g of *Nicotiana tabacum* leaf powders from Wannan District was placed in the container, wherein 60 volumes of water were added to reach a solid/liquid ratio of 1:60. The mixture was then subjected to sonication at a power of 200 W at 45 °C for 20 min. The obtained solution was then centrifuged at 10,000× *g* for 15 min at 4 °C, with supernatant collected for ethanol precipitation with three times the volume of pure ethanol. After protein removal using a Sevage reagent, the remaining ingredients were applied to a subsequent precipitation with three times the volume of pure ethanol and dialysis in the ethanol–water solution for 24 h, resulting in the crude PAP with an optimal yield of 3.15% (wet mass yields). The crude extracts of the PAP were used for the following animal studies, while it was purified for further characterization.

The crude extracts were then evaporated in air and lyophilized to obtain the powdered PAP. This was then subjected to purification by DEAE-52 cellulose chromatography (2.6 × 25 cm, Solarbio, Beijing, China). The elutes were obtained by a gradient elution using 0, 0.1, 0.2 and 0.3 mol/L NaCl in a sequential way. The elutes from the same peak were then merged, dialyzed in water and lyophilized to obtain the purified PAP.

### 2.2. Structural Characterization of the PAP

The purity of the PAP was first examined by UV spectral analysis. We prepared a 1 mg/mL purified PAP solution using a 752S spectrophotometer (Lengguang, Shanghai, China) to detect the absorption peaks at around 280 nm and 320 nm in the ultraviolet array range of 185 to 400 nm.

For structural analysis, 1 mg of purified PAP was mixed with dry KBr powder at a ratio of 1:100, which was then ground thoroughly and placed in a compressed slide. The preparations were then subjected to an infrared spectrometer (Nicolet, Thermo, Waltham (MA), USA) for scanning analysis in the range of 600 to 4000 cm^−1^. The combination of the identified absorption peaks could then be used to characterize the properties of the studied polysaccharide.

HP-GPC (High-Performance Gel Permeation Chromatography) was carried out to further characterize the extracted PAP. Ultrahydrogel 2000 and Ultrahydrogel 500 (Waters, Milford, CT, USA) were used in tandem to perform the gel permeation chromatography. The purified PAP was prepared to a concentration of 1 mg/mL and centrifuged at 11,000× *g* for 15 min. The obtained supernatant was passed through the 0.22 μm filter before being subjected to the columns, as mentioned above. The following conditions were set to conduct this experiment: an E2695 system (Waters, Milford, CT, USA), mobile phase: deionized water passed through a 0.22 μm filter; flow rate: 0.5 mL/min; acquisition volume: 20 μL; and a detector: differential refractive index detector.

### 2.3. Animals and Study Design

C57BL/6J mice were obtained from the Laboratory Animal Center of Anhui Medical University. All animal manipulations were approved by the Animal Committee of Hefei University of Technology, China. The offspring were randomly regrouped after weaning, and all the treatments were performed postnatally. Then, 100 ppm of lead acetate was administered from lactation to the behavioral test at PNW8 (postnatal week 8). The administration was conducted through dams during lactation and by drinking water ad libitum after weaning. The oral gavage of the PAP (200 ppm or 400 ppm, dosages used according to the pre-selection of prior experiments (unshown here); 400 ppm is equivalent to approximately 2.9~4 mg/kg body weight) was only performed after weaning, while each treatment was separated spatiotemporally from lead exposure to avoid direct contact. Tocilizumab with a concentration of 8 mg/kg was intraperitoneally injected into the lead-exposed mice once a week for three weeks before the behavioral test in the presence or absence of the PAP (400 ppm) treatment, to interfere with the expression of IL-6. Only female pups were selected for the subsequent measurements as the male pups did not display the uniform behaviors manifesting the memory impairments, as revealed by a previous study [25], as well as a previous finding [26] that females are more susceptible to postnatal lead exposure than their male counterparts. The diagram for the guidelines of this section is shown below.


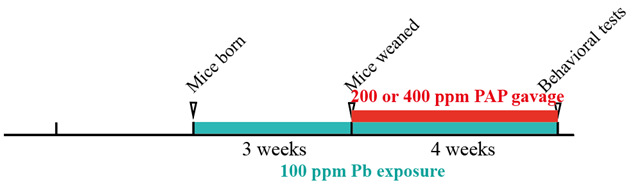


### 2.4. Morris Water Maze and Y-Maze Test

An animal behavioral assessment was conducted and completed during PNW8~9, as referenced previously [25], and the animal tracks were recorded and automatically analyzed by Smart tracking software (ANY-maze (version 7.45); Stoelting, Shanghai, China). Before the behavioral test, fecal samples were collected, and after the test, the serum and brain samples were collected, all of which were then subjected to the following analysis.

The Morris water maze (MWM) test was carried out in a circular water pool (400 mm deep) with a diameter of 1600 mm and a depth of 700 mm. The ambient temperature was constantly kept at 25 °C during the habituation, training and final test. After 5 days’ training in finding the hidden platform, the platform was eventually withdrawn on the test day (day 6), and each mouse was given a 90-s range of time to locate the orientation of the removed platform. The time spent and distances covered in the target area were then counted to represent the memory retention for each mouse. Moreover, the total movement of each mouse was also analyzed throughout the test procedure, to assess the influence of varying treatments on locomotive activity. A control polypeptide used here was a new *Dendrobium officinale* polysaccharide, which was a gift from our colleague from Hefei University of Technology.

The Y-maze test was performed as follows: at the beginning of each experimental session, each mouse was placed on the central platform. The subject mouse was then allowed to explore all three arms of the maze, and the number of spontaneous alternations (defined as the number of successive triplet entries into each of the three arms without any repeated entries) was monitored in a 10 min test session. The percentage of spontaneous alternation was calculated as the ratio of “the number of alternations” in the “total number of arm entries-2”, which was used to manifest the working spatial memory.

### 2.5. Microbiome Sequencing and Data Analysis

Next, 16S rRNA sequencing was used to characterize the gut microbiota of mice in response to treatments. Briefly, the fresh feces were collected before the behavioral test, mixed with saline solution and homogenized. The extracted DNA was subjected to 16SV4 PCR amplification, resulting in the DNA products being subjected to IonS5TMXL (ThermoFisher, Beijing, China) for sequencing. The data derived were further handled by the consecutive step of data splitting, filtration, chimera removal, OTU retrieval, annotation, phylogenetic construction and data normalization.

Differences in the gut microbiota between groups were first analyzed by phylogeny-based unweighted UniFrac distances, with the relative abundance of each phylum presented by a stacked column. R software (Version 4.0.3) was used to analyze the inter-group differences of beta diversity, and PCA graphs were invented through OriginPro (2023b, Northampton (MA), USA). LEfSe analysis was performed with the default filter value of 4.0.

### 2.6. Fecal Microbiota Transplant

Microbiota transfer was conducted as previously described [25] with some modifications. Specifically, microbiota from “Pb” or “Pb + 400PAP” donor groups were freshly harvested, and the fecal content was pooled, homogenized in a 1:4 ratio in sterile solution (1× PBS: 80% glycerol, ratio 1:1), and centrifuged at 300× *g* for 3 min, and the supernatant was collected and aliquoted. The fecal transplantation of “Pb-treated” mice (200 μL cocktail per mouse per day) began at PNW6 via gavage administration and lasted for two weeks, before they were subjected to behavioral assessment.

### 2.7. qPCR Analysis

The serum samples were used to profile cytokine expression by qPCR analysis, as mentioned previously [27]. Total RNAs were first extracted using the EasyPure RNA Kit (TransGen, Beijing, China). Subsequently, the reverse transcription was performed using a Reverse Transcription Kit (TransGen, Beijing, China). The qPCR mixture (20 μL) was comprised of 10 μL SYBR^®^ Premix Ex Taq, 0.8 μL each primer, and 2 μL template cDNA. qPCR was carried out using the standard 40-cycle conditions in LightCycle96 (Roche, Shanghai, China). The relative level of each transcript was quantified using GAPDH (glyceraldehyde-3-phosphate dehydrogenase) as an internal control. The primers used in this study are listed in Table 1.

### 2.8. Western Blotting

Since serum cytokine levels were found to influence the brain epigenetic molecules in the context of Pb-led memory impairment [25], the relative level of H3K27me3 was then quantified by Western blotting in the hippocampal tissues. The Western blotting was carried out as previously described [28]. Briefly, the mice were euthanized by cervical dislocation, with their hippocampus tissues subsequently separated, homogenized on the ice buffer and dissociated for 1 h. The cell lysates were then subjected to SDS-PAGE, and Western blotting was performed as previously described [29]. During the immune-binding stage, an anti-H3K27me3 antibody (Abcam, Shanghai, China, 1:1000) was used as the primary antibody, and densitometry was assessed with Image J software (NIH, Bethesda MD, USA), with band density normalized to GAPDH. At least three experiments were conducted before being subjected to analysis.

### 2.9. Statistical Analysis

Graph data were presented as means ± SEM. Statistical analysis was performed using Graphpad Prism (version 8.0) or OriginPro software (version 2023b). One-way ANOVAs with post hoc comparisons were used to analyze the multiple comparisons, while unpaired *t*-tests were used to compare two treatment groups. PCA was performed and presented using the Origin Pro software. The number of repeats involved in the respective analysis is shown in the corresponding figure legends.

## 3. Results

### 3.1. The Purified PAP Is a New Polysaccharide with β-Anomeric Carbon

**Figure 1:**
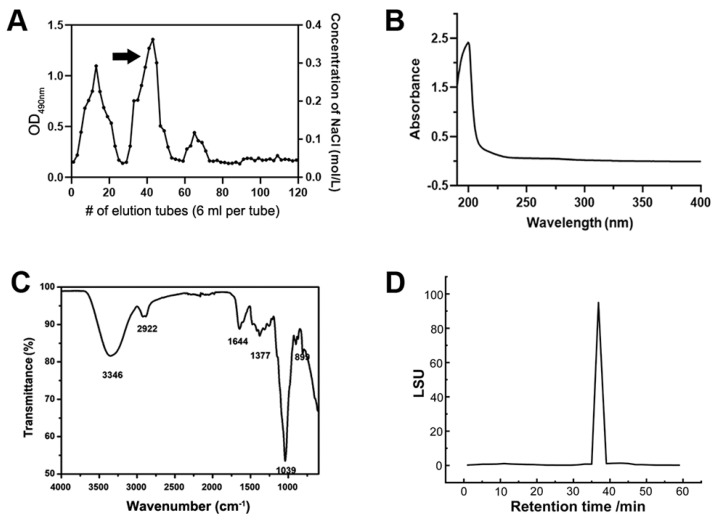
Purification and structural analysis of the PAP. (**A**) The elution chart of the DEAE-52 chromatography, which was used here to obtain the purified polysaccharide. The arrow indicates the absorption peak of the purified PAP. #, the number of elution tubes. (**B**) The UV spectral analysis of the purified PAP. (**C**) The ultrared spectral analysis of the purified PAP. The peaks at specific wavelengths might represent the specific bond types. (**D**) The PAP was further characterized by passing through the HP-GPC (High-Performance Gel Permeation Chromatography). LSU, light scattering units.

To date, there are only a very few active polysaccharides isolated from the *Nicotiana* leaves, except for Fr-I and Fr-II [17]. In order to gain the new form of plant active polysaccharide, ultrasonics were used to extract the crude polysaccharides, reaching a maximum yield of 3.15%. Subsequently, assisted by the DEAE-52 column chromatography, a new purified form of PAP was finally obtained. According to the elution profiles, the peak with the largest volume was subjected to collection, which also showed a clear boundary with other ingredients (Figure 1A). Its purity was further validated by UV spectrum analysis (Figure 1B), showing that no apparent peaks were identified to represent proteins or nucleic acids but instead represented sugar-like ingredients. Considering its structural properties, the isolated PAP was then subjected to ultrared spectrum analysis. As revealed by the results (Figure 1C), a strong absorption band at around 3346 cm^−1^ indicates the presence of hydroxyl groups, and a peak around 1039 cm^−1^ indicates the presence of a C-O band, suggesting the characteristic combinations of a sugar-like structure. In addition, the absorption band at 899 cm^−1^ is likely representative of the presence of the β-anomeric C1. In addition, the PAP was further subjected to HP-GPC analysis, and the detective chromatograph showed that there are no obvious peaks of other compounds detected around the major compound, probably indicating that the sample is a narrow-population polysaccharide (Figure 1D). In all, these absorption peaks indicate that a new form of polysaccharide was obtained. 

### 3.2. The PAP Shows Neuroregulatory Effect by Attenuating the Memory Deficit

**Figure 2:**
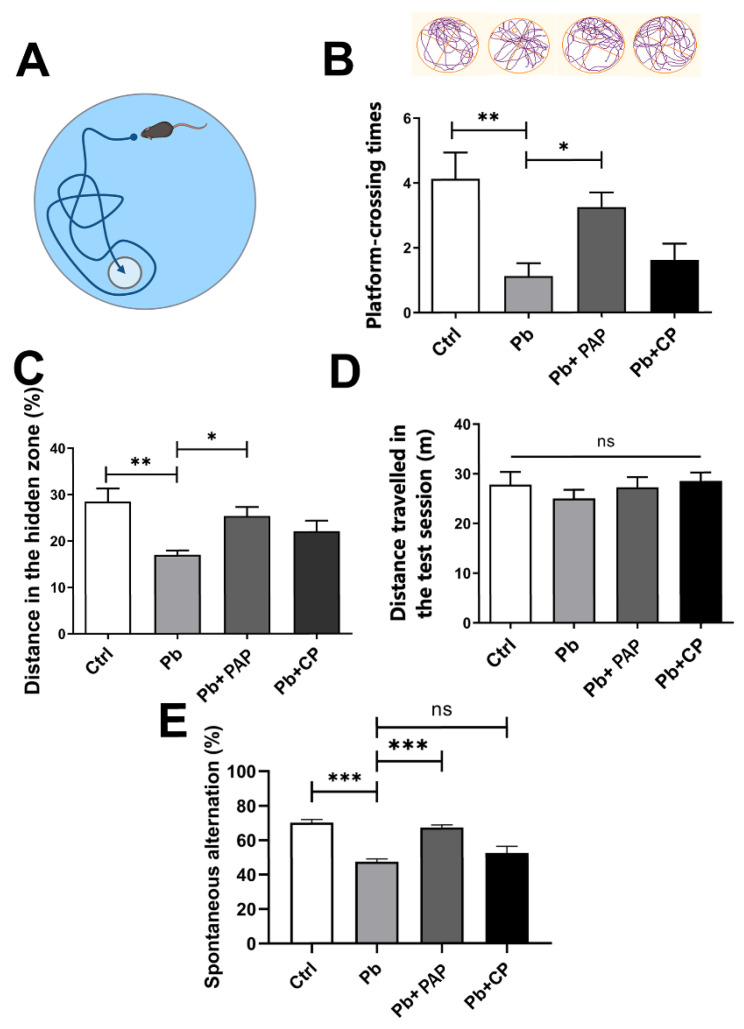
Morris water maze test of mice in response to lead and PAP treatment (*n* = 8–10). (**A**) The schematic illustration of the behavioral test paradigm. The ability of mice to locate the hidden platform is representative of spatial memory level. The test mouse was subjected to a five-day training session in a water maze to find a platform in a fixed position. At day 6, the platform was withdrawn, and the memory of the test mouse was then tested through their overlapped moving tracks with the position of platform or target quadrant. (**B**) Number of platform-crossings in the test session as well as the representative moving tracks. (**C**) Distance travelled in the target quadrant expressed as the ratio towards the total distance. (**D**) The total locomotive distance covered during the test session. (**E**) The alternation ratio of mice involved in the Y-maze test. The 400 ppm PAP was administered each day from weaning to behavioral assessment. Statistical analysis was performed using one-way ANOVA. All data are expressed as mean ± SEM. ns, *p* > 0.05; * *p* < 0.05; ** *p* < 0.01; *** *p* < 0.001. PAP, plant active polysaccharide; CP, a control plant polysaccharide *Dendrobium officinale*.

Despite the identification of the neuroregulatory activity of some plant polysaccharides, no similar effect was previously discovered with regards to active polysaccharides from *Nicotiana*. To explore this possibility, a memory deficit model was established through persistent postnatal lead exposure. As demonstrated by the Morris water maze (Figure 2A–C), lead exposure caused remarkable damage to the spatial memory, as the test mouse tended to delay or scramble in finding the hidden platform present in the first 5 days’ training sessions. However, when the PAP (400 ppm) was administered from weaning, the memory impairment was alleviated to a variable extent, with respect to either platform-crossing times (Figure 2B, *p* = 0.0475, One-way ANOVA) or distances covered in the target zone (Figure 2C, *p* = 0.0408, One-way ANOVA). It is noteworthy that this act does not apply to the ordinary plant polysaccharide, as a control polysaccharide did not produce a similar effect to the PAP (Figure 2A–C). CP was used here to study if the related benefits could be provided by an ordinary polysaccharide independent of the structural specificity. This result denied this hypothesis, demonstrating the importance of the specific species of PAP. In addition, no significant variations were observed in the overall movement of mice during the trial (Figure 2D), suggesting that the behavioral outcomes were not induced by the overall locomotion but the murine capacity of retrieving their spatial memory. Similar performance was also exhibited in a working spatial memory paradigm Y-maze test (Figure 2E, *p* < 0.001, One-way ANOVA), showing that the PAP attenuates the memory impairment. Collectively, the PAP rescues the memory impairment caused by lead exposure.

### 3.3. The PAP Reprograms the Gut Dysbiosis Accompanied by Memory Loss

**Figure 3:**
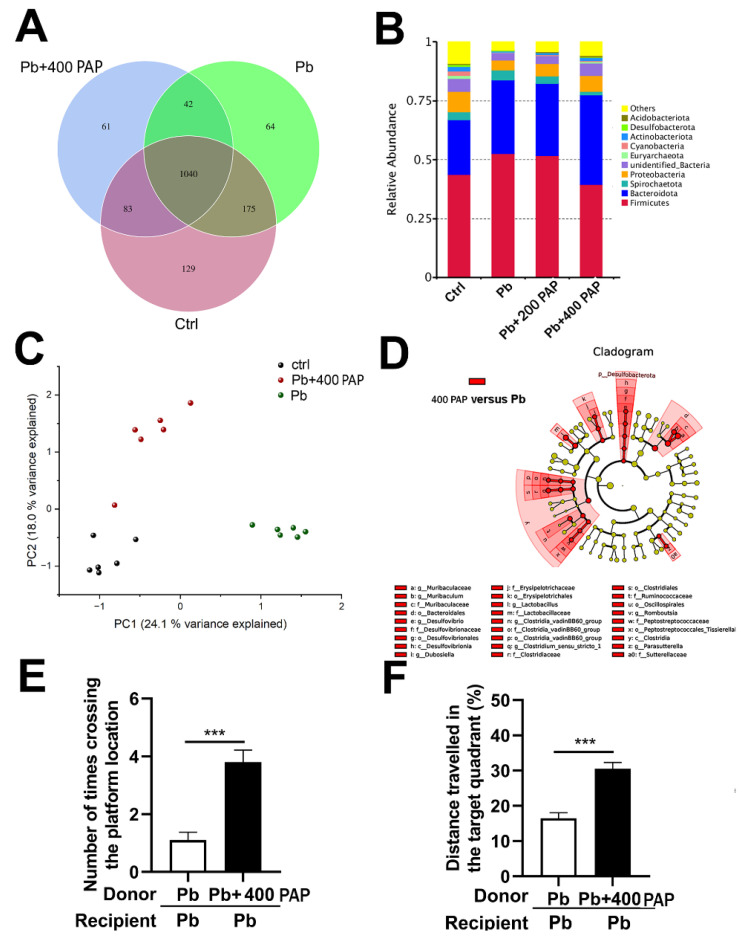
Gut microbiota analysis in response to lead and PAP treatment (*n* = 6). (**A**) Venn diagram depicting the number of species and common species identified in different treatment groups. (**B**) represents the stacked phyla compositions of the gut microbiota. (**C**) represents the PCA analysis of microbiota upon treatment of lead or 400 ppm PAP. (**D**) Microbial taxa identified using LEfSe analysis (LDA score > 4.0, *p* < 0.05) from the PAP + Pb group relative to the lead-exposed group, and the cladogram was shown on the left. The prominent taxa in the respective group were then listed below under the comparison of 400PAP vs. Pb. (**E**) Number of platform-crossings in the test session for the fecal microbiota transplant (FMT) experiment (*n* = 7). FMT was carried out by continuously transferring the microbiota from donors to the corresponding recipients. The procedure started at PNW6 and lasted for two weeks before behavioral evaluation. (**F**) Distance travelled in the target quadrant expressed as the ratio to the total distance. The donors and recipients involved were indicated beneath the graph. PAP, plant active polysaccharide. *** *p* < 0.001.

The gut microbiota is closely associated with brain activity through the gut–brain axis. In our memory deficit model, the gut microbiota was also disrupted in a profound way, as exhibited by the overall architecture of the microbiota residing in the test mice (Figure 3A–D). This dysbiotic state is consistent with the previously proposed link between lead exposure and the gut microbiota [25]. With the addition of the PAP (400 ppm), a proportion of taxa was resumed or identified in this microbial community (Figure 3A). At the phylum level, the PAP caused a shift in microbial structure in an overall way, manifested by the reduced abundance of Firmicutes as well as the enhanced presence of Bacteroidetes. As a result, the disrupted F/B ratio was partly restored by the PAP—a phenomenon representative of a restoration of the normal and balanced microbiota. Of note, the microbiota-modifying effect of the PAP is dose-dependent, as a low dose (200 ppm) did not provide a comparable consequence (Figure 3B), but it is noteworthy that this dosage could only be reflected by the relative abundance of some phyla instead of other detailed taxa. Moreover, PCA results showed an interesting tendency: the addition of the PAP drove the microbiota to a state at which the composition was closer to the untreated mice than the lead-exposed conspecifics (Figure 3C). LEfSe analysis revealed a collection of taxa standing out in the PAP-treated group relative to the memory loss group (Figure 3D). These enriched bacteria included some promiscuous taxa like Muribaculum, Desulfovibrio, Lactobacillus, etc., which might suggest that the keystone strains were resuming dominance in the PAP-treated microbiota. Subsequently, we carried out the fecal microbiota transplant (FMT) experiments. When the microbiota from the PAP-intervened group was transferred to the Pb-exposed donors, it was found that the murine behaviors in the Morris water maze were restored remarkably, with respect to the number of times the test mouse crossed the withdraw platform (Figure 3E) or the distance travelled in the target quadrant relative to the total distances (Figure 3F), which was in contrast to the Pb-exposed donors. These data provide direct evidence that the PAP-modifying microbiota plays important roles in the studied gut–brain axis as well as the ensuing spatial memories. Taken together, the PAP reshapes the gut microbiota disrupted by lead exposure.

### 3.4. IL-6 Is Implicated in the PAP-Triggered Gut–Brain Communication

**Figure 4:**
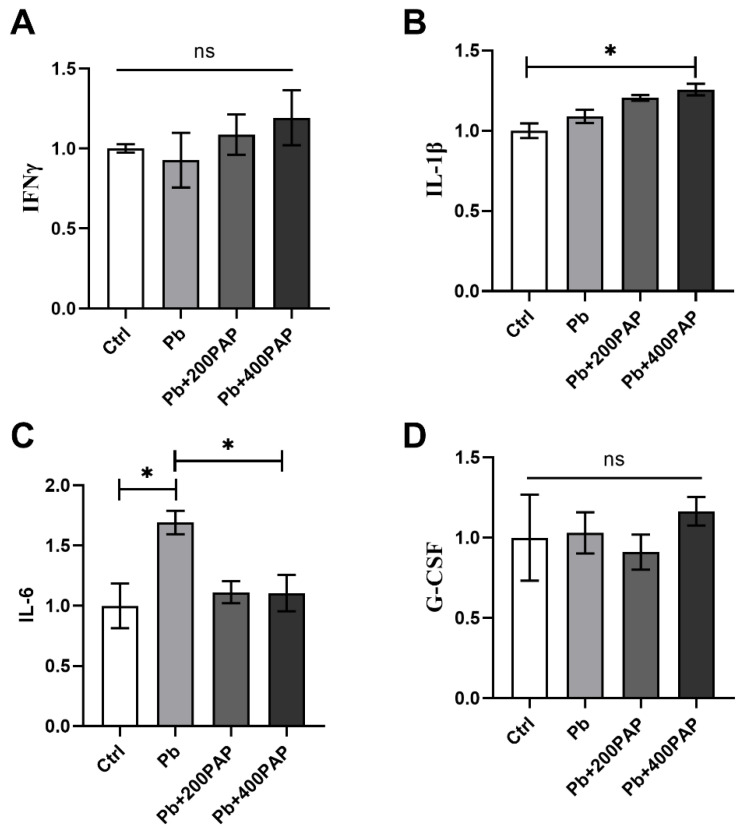
Cytokine profiling in response to lead and PAP treatment (*n* = 4). (**A**–**D**) The mice sera were collected following sacrifice, and their mRNA were extracted, reverse transcribed and subjected to qPCR analysis. In terms of cytokines, IFNγ (**A**), IL-1β (**B**), IL-6 (**C**) and G-CSF (**D**) were quantified and normalized against the untreated group. The 200 or 400 ppm PAPs were administered to the lead-exposed mice prior to measurement. Statistical analysis was performed using one-way ANOVA. All data are expressed as mean ± SEM. ns, *p* > 0.05; * *p* < 0.05. PAP, plant active polysaccharide.

Since the PAP can trigger a series of changes in brain behavior and the gut microbiota, it is then required to decipher if a specific gut–brain axis is involved in this cascade. The immune pathway has long been considered one of the major routes connecting the microbiota and brain [30]; thus, plasma cytokines were first profiled in the studied physiological settings. According to the results, lead exposure did cause the diversified expressional alteration of some cytokines (Figure 4A–D). Except for IL-6 (*p* = 0.018 between Ctrl and Pb; *p* = 0.048 between Pb and PAP, One-way ANOVA), no uniform variations were detected along with the administration of the PAP, suggesting that IL-6 might be involved in the PAP-led improvement. Moreover, the reduction of IL-6 did not vary depending on the dosages of PAPs used.

**Figure 5:**
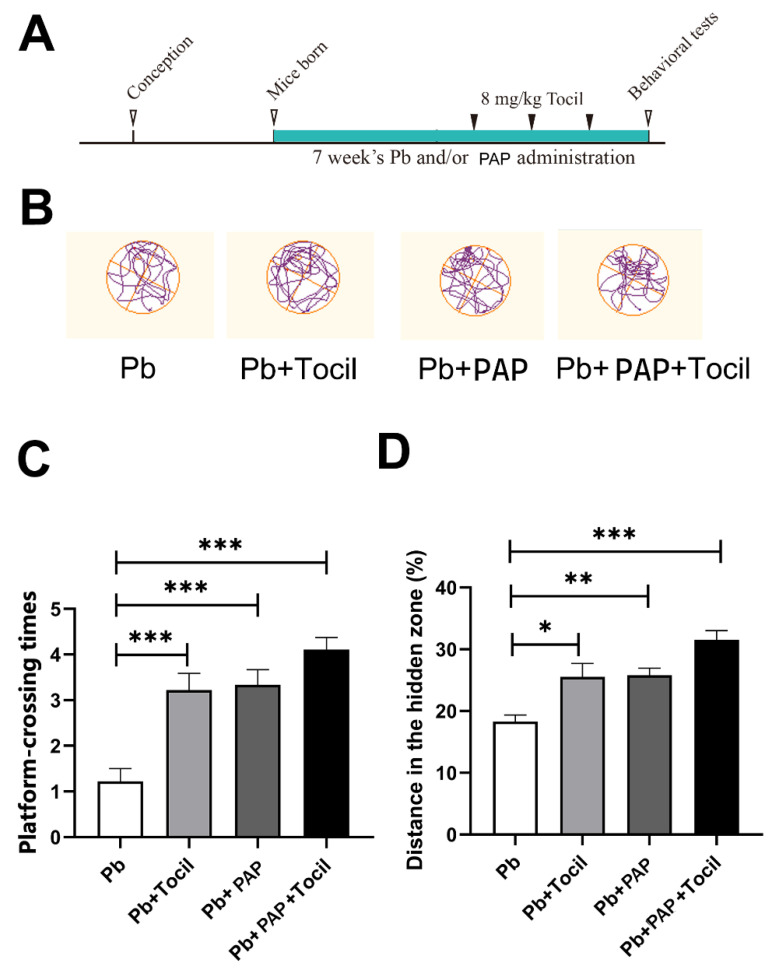
Morris water maze test of mice in response to Tocilizumab intervention (*n* = 8). (**A**) Timeline of Tocilizumab intervention paradigm. 8 mg/kg Tocilizumab (an IL-6 antagonist) was intraperitoneally injected to the mice with lead exposure once in a week from weaning to behavioral test. A new group was set as both the PAP and Tocilizumab were administered three weeks before behavioral assessment. All mice were then subjected to the Morris water maze test at PNW8~9. (**B**) Representative moving tracks of the test mice upon various treatments. (**C**) Number of platform-crossings in the test session. (**D**) Distance travelled in the target quadrant expressed as the ratio towards the total distance. Statistical analysis was performed using unpaired *t*-test. All data are expressed as mean ± SEM. * *p* < 0.05; ** *p* < 0.01; *** *p* < 0.001. PAP, plant active polysaccharide. Tocil, tocilizumab, an antagonist of IL-6.

To further dissect the roles of IL-6, a related receptor antagonist, Tocilizumab, was i.p. injected into the mice from weaning to adulthood. As shown by the behavioral assessment (Figure 5B–D), the platform-crossing times (*p* = 0.0013, One-way ANOVA) and distances (*p* = 0.0094, One-way ANOVA) were significantly increased due to the use of Tocilizumab, indicating that the memory loss was ameliorated by the IL-6 inhibitor—an effect in parallel with the PAP. Moreover, when the PAP and Tocilizumab were jointly administered, there appeared a tendency for the behavioral performance to further improve (*p* < 0.001 between Pb and Pb + PAP + Tocil, One-way ANOVA), strengthening the negative relationship between IL-6 and behavior. In line with the concerted changes of IL-6 level under PAP treatment, we might conclude that the PAP plays neuroregulatory roles through adjusting IL-6 levels.

### 3.5. H3K27me3 Is Responsive to the Studied Gut–Brain Axis Evoked by the PAP

**Figure 6:**
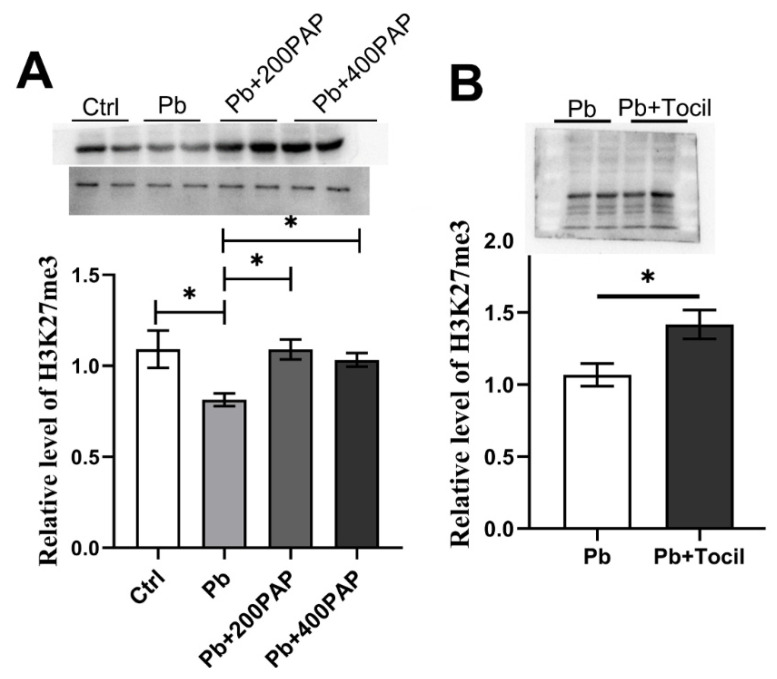
Relative protein level of H3K27me in hippocampus of mice with the lead, the PAP or Tocilizumab treatments. (**A**) Representative Western blotting graphs as well as their relative densities of H3K27me3, which was studied with respect to hippocampus of mice with lead and/or the PAP (200, 400 ppm) treatment. The lower band is representative of the blotting of internal control GAPDH. (**B**) Western blotting graphs as well as protein levels of H3K27med, in response to Tocilizumab intervention. Statistical analysis was performed using one-way ANOVA or unpaired *t* test. All data are expressed as mean ± SEM from three experiments. * *p* < 0.05. PAP, plant active polysaccharide; Tocil, tocilizumab, an antagonist of IL-6.

As an epigenetic mark, H3K27me3 was previously found to be central in receiving gut signals and modulating spatial memory [25]. In order to further study the molecular mechanisms surrounding the PAP-mediated intervention, cerebral tissues were collected from the sacrificed mice treated with lead or the PAP. As revealed by Western blotting densities, lead exposure decreased the protein level of H3K27me3 in the hippocampus section—a trend that was further reversed by either the 200 or 400 ppm PAP (Figure 6A). These data might indicate that H3K27me3 is involved in the studied intervention. Furthermore, when tocilizumab was administered, a similar alteration arose again with respect to hippocampal H3K27me3 (Figure 6B, *p* = 0.033), suggesting that this epigenetic molecule is correlative with the concentration of plasma IL-6. In summary, H3K27me3 is responsive to the PAP-mediated improvement of memory lesions.

## 4. Discussion

In this study, a new form of active polysaccharide was identified from *Nicotiana* leaves and functionally characterized. More specifically, its neuroregulatory function was manifested by the attenuation of memory deficits, via a gut–brain pathway specifying the microbiota/IL-6/H3K27me3. This is an interesting example of a new form of plant active polysaccharide, which was functionally characterized in terms of gut–brain-modulating activity. This suggests that the PAP has the capacity to adjust or improve neural activity, expanding its potential application in the related field. Given the specificity of the structure–function relationship, however, this finding could not be used as plausible evidence to deduce the biological activity of other PAPs isolated in future studies. As PAPs have been applied in the food industry as emulsifiers, edible film packaging or dietary ingredients [1,2], their health-promoting effects are supposed to promote their functionality and feasibility in the field of food processing or functional foods.

There are some instances of active polysaccharides from plants modulating brain behavior in distinct sets of pathways. For example, *Astragalus* polysaccharides are able to attenuate the neuroinflammation and balanced gut microbiota in mice challenged by *Salmonella* [31]; the stigma maydis polysaccharide can have a positive effect on the autistic performance of VPA-exposed rats [32]; and a proprietary mixture of non-starch polysaccharides led to acute behavioral improvement in healthy middle-aged adults with mental fatigue [33]. According to these reports, different polysaccharides may have unique influences on the specific aspect of central nervous system as well as its related behavioral outcomes. This specificity is supposed to be correlated with the molecular structure of each compound per se. In the current case, the PAP is a polysaccharide with β-anomeric carbon, which might be associated with memory-modulating activity based on this study. It is noteworthy that this function should not be considered universal within the range of plant polysaccharides, as the controlled polysaccharide used here did not have a prominent effect (Figure 2), as well as the reported mixture of polysaccharides, which showed a better protection compared to sucrose [33]. To date, it remains a challenging task to predict the function of a polysaccharide from its structural features, let alone the neuroregulatory activity. This limitation also applies to the current study, whereas the purification and basic characteristics of new PAPs were provided in the absence of more detailed structural characteristics like the sugar compositions, molecular range, etc. Therefore, further efforts should be made to establish a range of structure–function relationships before offering a bona fide mechanistic insight into using specific structures to predict neuroregulatory activities. That makes it important to gain more data concerning the function of a specific polysaccharide based on experimental results, which can then lay the foundation for metadata analysis.

The PAP reshaped the gut microbiota of mice with memory deficits. It is not a rare incident for a plant polysaccharide to modify microbial structure. In the event of *Astragalus*, the sugar-like substance led to the upregulation of lactobacillus, as well the downregulation of Bacteroidetes and Enterobacteriaceae [31]; for stigma maydis, Prevotellaceae and Lachnospiraceae_NK4A136_group were increased after its intervention [32]. We cannot find a significant relevance of these changes with the PAP-induced microbiota restructuring, which exhibited the enhanced presence of a range of probiotic or keystone taxa, like Muribaculum, Lactobacillus, etc. Of interest, these bacteria are normally regarded as short-chain fatty acid producers or as anti-inflammatory [34,35]. This notion is consistent with the current finding that IL-6, a proinflammatory cytokine, was inhibited by the reshaped microbiome, and thus conveyed the relevant signal to the brain. IL-6 seems crucial in this gut–brain process related to the lead-led neural damage—a finding consistent with the prior finding [25] that IL-6 has a positive relationship with associated memory deficits. Still, it remains elusive which key bacteria plays a primary role in stimulating the production of IL-6 in the circulating system, because unlike the prior study, we did not find significant variations of Bifidobacterium, Actinobacter or Fibrobacteria in response to the PAP intervention. This probably suggests the action of multiple bacteria instead of a specific taxon in relation to IL-6 stimulation. As IL-6 is considered the messenger conveying the gut-derived signal to the brain, the intraperitoneal injection of Tolizumab is not the sole or optimal route to conduct the intervention. Another limitation here is the dosage associated with this antagonist, and the dose–response curve is not clarified in the current study. However, it needs to be stressed that there might be a series of confounding factors that have impact on the gut microbiota in addition to the studied polysaccharide, like diet, inbred group, cultivation conditions, and so on. Therefore, caution should be taken to interpret the relationship between the gut microbiota and the behavioral consequences in the brain.

H3K27me3 is a relatively stable histone mark. In post-mitotic neurons, H3K27me3 has an appropriate function of maintaining cell identity via long-lasting gene repression [13]. Our results suggest that H3K27me3 is correlative with IL-6 changes—a finding consistent with its regulatory function in the spatial memory behavior [36]. Therefore, the PAP harbors the capacity to exert effects on this epigenetic mark, probably through the gut–brain axis featuring IL-6. These data might show a link between plant polysaccharides with complex histone marks in the brain, establishing a new perspective in the health-promoting function of this species of biomolecules. Furthermore, the fact that H3K27me3 is also reversible may underlie the microbiota-oriented strategy against cognition-related psychiatric diseases. Furthermore, the gut–brain axis might not be the only inter-organ route mediating the brain-modulatory effect of the PAP, while the roles of the liver–brain axis still warrant further investigations.

## 5. Conclusions

This study was conducted to isolate and functionally characterize a new plant active polysaccharide. Memory-modulating activity was achieved by the PAP-modifying microbiota, as well as the ensuing gut–brain axis centering at IL-6. This study offers a functional link between neuromodulation and PAPs and clarifies the specific gut–brain mechanism in the context of PAP intervention. These findings may indicate an emerging research direction for plant polysaccharides, paving the way for the advanced utilization of bioactive compounds from this species of economic plant. As neurological impairment or deficits are severe health issues, the neuroregulatory function of this new polysaccharide might offer perspectives for potential pharmaceutical interventions in the future.

## Figures and Tables

**Table 1:** Primers used in this study.

Primer	Sequence	Gene
IL-6 477F	AAGCCAGAGTCATTCAGAGCAA	IL-6
IL-6 635R	GGATGGTCTTGGTCCTTAGCC	
IFN-γ 377F	ACAACCCACAGATCCAGCACAA	IFN-γ
IFN-γ 482R	AATCAGCACCGACTCCTTTTCC	
GCSF 805F	TGGAGGGCAGGGAAGGAGATA	GCSF
GCSF 1063R	CGGGGTCAGGAAAACCTACAAC	
IL-1b 54F	GCTATGGCAACTGTCCCTGAAC	IL-1β
IL-1b 225R	CGAGATGCTGCTGTGAGATTTG	

## Data Availability

The data presented in this study are available on request from the corresponding author (accurately indicate status).
